# Testicular cancer follow-up costs in Germany from 2000 to 2015

**DOI:** 10.1007/s00432-021-03643-1

**Published:** 2021-04-22

**Authors:** Thomas Michaeli, Julia Michaeli, Daniel Michaeli

**Affiliations:** 1 Fifth Department of Medicine, University Medical Centre Mannheim, Heidelberg University, Theodor-Kutzer-Ufer 1-3, Mannheim, 68167 Germany; 2 Department of Obstetrics and Gynecology, Asklepios-Clinic Hamburg Altona, Asklepios Hospital Group, Hamburg, Germany

**Keywords:** Testicular cancer, Follow-up, Cost, Health insurance, Medical imaging

## Abstract

**Purpose:**

Advances in testicular cancer screening and therapy increased 10-year survival to 97% despite a rising incidence; eventually expanding the population of survivors requiring follow-up. We analyzed 10-year follow-up costs after testicular cancer treatment in Germany during 2000, 2008, and 2015.

**Methods:**

Testicular cancer follow-up guidelines were extracted from the European Association of Urology. Per patient costs were estimated with a micro-costing approach considering direct and indirect medical expenses derived from expert interviews, literature research, and official scales of tariffs. Three perspectives covering costs for patients, providers, and insurers were included to estimate societal costs. Cost progression was compared across cancer histology, stage, stakeholders, resource use, and follow-up years.

**Results:**

Mean 10-year follow-up costs per patient for stage I seminomatous germ-cell tumors (SGCT) on surveillance declined from EUR 11,995 in 2000 to EUR 4,430 in 2015 (*p* < 0.001). Advanced SGCT spending shrank from EUR 13,866 to EUR 9,724 (*p* < 0.001). In contrast, expenditure for stage II SGCT increased from EUR 7,159 to EUR 9,724 (*p* < 0.001). While insurers covered 32% of costs in 2000, only 13% of costs were reimbursed in 2015 (*p* < 0.001). 70% of SGCT follow-up resources were consumed by medical imaging (x-ray, CT, ultrasound, FDG-PET). Spending was unevenly distributed across follow-up years (years 1–2: 50%, years 3–5: 39%, years 5–10: 11%).

**Conclusions:**

The increasing prevalence of testicular cancer survivors caused German statutory insurers to cut per patient cost by up to 80% by budgeting services and decreasing reimbursement rates. The economic burden was gradually redistributed to patients and providers.

## Introduction

Germany spent 6.8% of the gross domestic product on cancer in 2014, representing a 1% increase from 1995 (Böhm et al. 2009). Testicular cancer is considered the most common malignancy for men aged 25 to 45 years in Germany with an age-standardized incidence rate of 10.2 per 100,000 inhabitants per year (Robert Koch Institute 2020). A combination of early detection and treatment advances of testicular cancer led to improved 10-year survival rates of 97% in Germany (Robert Koch Institute 2020). Treatment strategies for metastatic disease depend on cancer histology [seminomatous germ-cell tumor (SGCT) vs. non-seminomatous germ-cell tumors (NSGCT)], disease dissemination, and further risk factors impacting prognosis. Treatment strategies can either have a curative or palliative intent.

Recently, health policy has focused on cancer prevention and treatment strategies. Scholars commonly investigate testicular cancer therapy options regarding their efficacy, side effects, and cost-effectiveness. Distinct follow-up treatment options are rarely reassessed for cost-effectiveness in Germany. However, authors come to different conclusions regarding the cost-effectiveness of follow-up strategies according to testicular cancer type and stage and analyzed year. Clasen et al. (2009) previously evaluated a follow-up strategy based on chest X-ray, abdominopelvic computed tomography (CT), abdominal ultrasound, and tumor markers for patients with stage I seminoma after treatment with radiotherapy (Clasen et al. 2009). Among the three examined options, abdominopelvic CT was the most cost-effective choice, even though it incurred highest costs. In contrast, Gietema et al. (2002) found that routine chest X-rays are not cost-effective during follow-up of patients after chemotherapy of metastatic NSGCT (Gietema et al. 2002). Early detection and treatment advances alongside enhanced imaging options for testicular cancer follow-ups were consequently incorporated into the European Association of Urology (EAU) guidelines.

A study evaluating follow-up costs under updated EAU guidelines for testicular cancer on a patient level in Germany is missing. Consequently, this research scrutinizes 10-year follow-up costs from 2000 to 2015 according to EAU recommendations. A detailed micro-costing approach considering direct and indirect medical expenses for insurers, providers, and payers is employed to estimate overall societal costs.

### Testicular cancer guidelines

The EAU issued and updated testicular cancer follow-up guidelines from 2000 to 2015 (Laguna et al. 2001; Albers et al. 2005, 2011, 2015; Krege et al. 2008a, b). Recommendations separate follow-up patterns depending on the identified histology, stage, and post-orchiectomy treatment alternatives.

#### Non-seminoma

The aim of follow-up for NSGCT is to detect relapses and contralateral neoplasia by performing regular examinations during the first 2 years. Depending on the initial treatment and on the stage, examinations were recommended in months 2, 4, 6, 8, 10, 12, 16, 20, 24, 30, 36, 42, 48, 54, 60, and thereafter annually until year 10. Regular examinations include a blood control for serum tumor markers lactate dehydrogenase, beta-human chorionic gonadotropin, and alpha-fetoprotein. Clinical or laboratory suspicion of cancer recurrence should be followed by a chest X-ray or abdominal computed tomography scan. Abdominal computed tomography scan is recommended in months 4, 8, 12, 18, 24, 36, 48, and 60. Patients with advanced diseases require more intense follow-ups and intensified medical imaging (Table 1).

**Table 1:** Annual frequency of recommended follow-up procedures for metastatic NSGCT and SGCT (stage III) according to EAU guidelines in 2000, 2008, and 2015

Follow-up year	EAU Guideline 2000						EAU Guideline 2008						EAU Guideline 2015					
	1	2	3	4	5	> 5	1	2	3	4	5	> 5	1	2	3	4	5	> 5
Physical examination	12×	6×	4×	3×	2×	1×	4×	4×	2×	2×	2×	1×	4×	4×	2×	2×	2×	1×
Tumor markers	12×	6×	4×	3×	2×	1×	4×	4×	2×	2×	2×	1×	4×	4×	2×	2×	2×	1×
Chest X-ray	12×	6×	4×	3×	2×	1×	4×	4×	2×	2×	2×	1×	4×	4×	2×	2×	2×	1×
Abdominopelvic CT^a^	–	–	–	–	–	–	2×	2×	1×	1×	1×	1×	2×	2×	1×	1×	1×	–
Chest CT^a^	–	–	–	–	–	–	–	–	–	–	–	–	1×	1×	1×	1×	1×	–
Brain CT^a^	–	–	–	–	–	–	–	–	–	–	–	–	1×	1×	1×	1×	1×	–

If available, FDG-PET/CT could be performed optionally in 2008 and 2015

*EAU* European Association of Urology, *CT* computed tomography, *FGG-PET/CT* fluorodeoxyglucose positron emission tomography/computed tomography

^a^Abdominopelvic, chest, and brain CT as indicated in year 2000 and 2008 according to histology and prior treatment

#### Seminoma

SGCT patients following radiotherapy are examined with tumor markers and chest x-rays four times in year one, three times in year two and three, and twice a year thereafter until year five. Medical imaging was recommended once per year until year two. In contrast, patients after chemotherapy are followed more regularly. Advanced stages are recommended to be seen regularly and might require a CT of the abdomen, pelvis, brain, and chest (Table 1).

#### EAU updates

The EAU revised guidelines by generally decreasing the amount of required physical examinations, blood tests, and chest X-rays in year 1 and 2, while increasing the amount of annually recommended imaging tests within the first 5 years (Table 1).

## Methods

Follow-up costs were projected for four distinct follow-up types for SGCT/NSGCT: stage I (post-radiotherapy/post-surgery and post-chemotherapy), stage I (surveillance), stage II, and stage III. As EAU guidelines focus on the first 10 years post-treatment, a 10-year time horizon was assumed appropriate. Cost data were derived with a micro-costing approach based on patient level data. The developed costing approach estimates follow-up costs from all three perspectives—payers, providers, and insurers—to then estimate the overall societal impact in 2000, 2008, and 2015.

### Payer perspective

The payer perspective assesses opportunity costs relevant for testicular cancer follow-ups. Time consumption was derived from literature and expert opinions (Table 2). Forgone opportunity costs were calculated according to yearly average salary and the productive hours. Calculations considered the higher annual income of privately insured patients (2000: EUR 39,574; 2008: EUR 48,150; 2015: EUR 54,900) relative to statutory insured patients (2000: EUR 30,612; 2008: EUR 37,236; 2015: EUR 43,344). The analysis considers the German ratio of 88% statutory and 12% privately insured patients.

**Table 2:** Input Parameters

	Private Health Insurance: GOÄ (EUR)			Social Health Insurance: EBM (EUR)			Time consumption (minutes)						
	2000	2008	2015	2000	2008	2015	Physician	References	Assistant	References	Patient	References	Distribution
Physician–patient consultation	20.10	20.10	20.10	9.86	20.33	20.34	7.6	Irving et al. (2017)	–		7.6	Irving et al. (2017)	Gamma
Physical examination	34.86	34.86	34.86	11.90	10.75	11.61	15.8	Irving et al. (2017)	–		15.8	Irving et al. (2017)	Gamma
Tumor markers (incl. blood taking)	35.66	35.66	35.66	26.50	13.45	12.75	–		5.0	Irving et al. (2017)	5.0	Irving et al. (2017)	Normal
Testicular sonography	26.81	26.81	26.81	14.88	8.23	8.94	20.0	Andreas (2020)	–		20.0	Irving et al. (2017)	Gamma
Chest X-ray	47.21	47.21	47.21	16.74	9.07	9.76	10.0	Alth and Vorbeck (2020), Andreas (2020), Schmitt (2020)	15.0	Radiologie Oberland (2020a, b)	10.0	Alth and Vorbeck (2020), Andreas (2020), Schmitt (2020)	Normal
Abdominal CT scan	272.79	272.79	272.79	155.50	124.49	136.41	21.1	U.S. National Library of Medicine (2020)	30.0	Plathow (2004)	31.1	U.S. National Library of Medicine (2020)	Normal
Pelvic CT scan	272.79	272.79	272.79	155.50	109.70	152.29	13.0	Hasson Aljebori and Yasen (2019)	30.0	Plathow (2004)	13.0	Hasson Aljebori and Yasen (2019)	Normal
Chest CT scan	241.31	241.31	241.31	79.50	109.37	120.12	10.0	Irving et al. (2017)	25.0	Plathow (2004)	10.0	Irving et al. (2017)	Normal
Brain CT scan	209.84	209.84	209.84	74.40	117.43	115.40	15.0	Radiologie Oberland (2020a, b)	20.0	Plathow (2004)	15.0	Radiologie Oberland (2020a, b)	Normal
FDG-PET/CT scan	–	1012.80	1012.80	–	–^a^	–^a^	30.0	Irving et al. (2017)	10.0	Irving et al. (2017)	30.0	Irving et al. (2017)	Normal
Travel time	–	–	–	–	–	–	–		–		60.0	Irving et al. (2017)	Gamma
Waiting period	–	–	–	–	–	–	–		–		45.0	Radtke (2019)	Gamma
Travel cost (km)	–	–	–	–	–	–	–		–		40.0	Randelhoff (2011)	Gamma
Parking cost	–	–	–	–	–	–	–		–		45.0	Randelhoff (2011)	Gamma

Reimbursement rates for private and statutory health insurance were extracted from the respective reimbursement catalog: “Gebührenordnung für Ärzte (GOÄ)” and “Einheitlicher Bewertungsmaßstab (EBM)” (Brück 1998; Kassenärtzliche Bundesvereinigung 2000, 2008, 2015). Time consumption for physicians, medical assistants, and patients was extracted from relevant literature and confirmed in expert interviews

*CT* computed tomography, *FGG-PET/CT* fluorodeoxyglucose positron emission tomography/computed tomography

^a^FDG-PET scan was not reimbursed by the German statutory health insurance in 2008 and 2015 for this indication

### Provider perspective

The provider perspective estimates opportunity costs for all relevant health care providers involved in testicular cancer follow-ups. In Germany, the provider perspective is relevant since budgeting and reimbursement restrictions lead to a discrepancy between the provider’s possibility to bill medical services and actual performed medical activities. Time consumption were again based on previous literature and expert interviews (Table 2). The forgone opportunity cost was calculated based on the average yearly salary and the productive hours (Netten and Curtis 2000; Curtis 2008; Curtis and Burns 2015). Opportunity costs per hour of patient contact rose throughout the examined period for both physicians (2000: EUR 203.20; 2008: EUR 230.25; 2015: EUR 305.37) and medical assistants (2000: EUR 46.13; 2008: EUR 40.28; 2015: EUR 59.32).

### Insurer perspective

The insurer perspective considers the bill that the provider hands to the insurer. Due to Germany’s dual insurance system, a separate costing approach was considered for both the social health insurance (SHI) and the private health insurance (PHI). SHI and PHI funds are charged according to reimbursement rates published in a regularly updated catalogs (Brück 1998; Kassenärtzliche Bundesvereinigung 2000, 2008, 2015). Reimbursement quotes for related (Table 2) services from both scales were derived for 2000, 2008, and 2015.

### Cost calculation

The frequency of recommended consultations, examinations, and diagnostic tests was extracted from EAU guidelines. Thereafter, the opportunity costs for physicians and patients, resulting from forgone time consumption for follow-ups, were calculated alongside the resulting expenditure bill for the insurance (Table 1). The hospital and community health services index was used to adjust all healthcare related costs for inflation. Cost progression was compared across years, stakeholder, and resource use.

### Sensitivity analysis

We conducted a probabilistic sensitivity analysis to account for variations in the length of physician–patient consultations, examinations, and diagnostic tests. Therefore, provider’s and payer’s time consumption parameters were drawn by random sampling from their defined distribution (Table 2). The analysis estimated costs for 1000 patients per treatment cohort and year. Cost data were expressed as means ± standard deviations. For the two-factorial analysis of variance, ANOVA with Dunnett’s test was applied. A two-tailed probability value < 0.05 was considered significant.

## Results

**Fig. 1:**
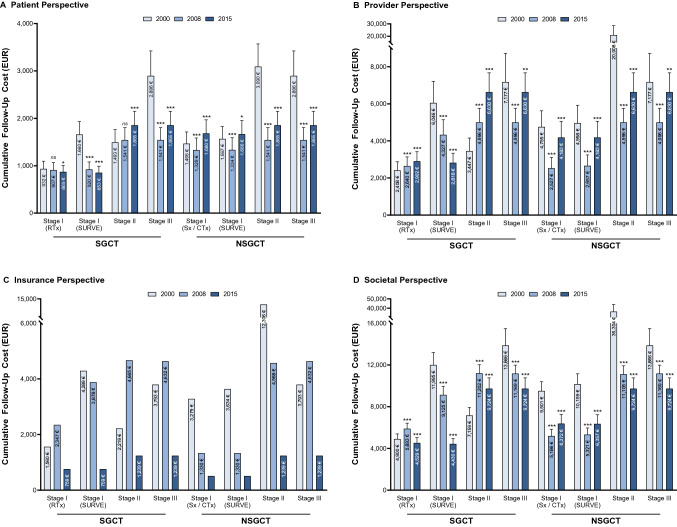
Cumulative 10-year testicular cancer follow-up costs (EUR) per patient by initial treatment type from the **a** payer, **b** provider, **c** insurance, and **d** societal perspective. All costs were inflation adjusted with the hospital and community health services index. *SGCT* seminomatous germ-cell tumor, *NSGCT* non-seminomatous germ-cell tumor, *RTx* radiotherapy, *SURVE* surveillance, *Sx* surgery, *CTx* chemotherapy. *p* values compared to year 2000: *p* < 0.05 (*), *p* < 0.01 (**), *p* < 0.001 (***). Bars show standard deviations. Insurance bills do not possess standard deviations because fixed reimbursement rates were extracted from the official scales of tariffs

Estimated 10-year prostate cancer follow-up costs ranged from EUR 4430 to EUR 36,304 per person (Fig. 1). However, costs progression diverged between payers, providers, and insurers from 2000 to 2015.

### Payer

10-year follow-up costs for payers decreased throughout the examined period (Fig. 1a). Follow-up costs for stage I SGCT after radiotherapy decreased from EUR 932 in 2000 to EUR 869 in 2015 (*p* < 0.05). The decline was even sharper for follow-up schedules for stage I SGCT after surveillance (2000: EUR 1660; 2015: EUR 853; *p* < 0.001) and with SGCT/NSGCT in stage III with metastasis (2000: EUR 2896; 2015: EUR 1855; *p* < 0.001). In contrast, follow-up cost for stage II SGCT surged from EUR 1493 in 2000 to EUR 1855 in 2015 (*p* < 0.001). Regarding NSGCT, follow-up costs for stage I after surgery/chemotherapy increased from EUR 1466 in 2000 to EUR 1680 in 2015 (*p* < 0.001).

### Provider

The calculated 10-year follow-up costs for providers showed varying patterns depending on cancer stage and histology (Fig. 1b). Costs after radiotherapy of SGCT in stage I surged from EUR 2408 in 2000 to EUR 2902 in 2015 (*p* < 0.001). Follow-up costs for SGCT in stage I on surveillance steeply declined (2000: EUR 6046; 2015: EUR 2818; *p* < 0.001), while SGCT in stage II surged (2000: EUR 3447; 2015: EUR 6630; *p* < 0.001). There was a decline in follow-up costs for advanced SGCT/NSGST in stage III from EUR 7177 in 2000 to EUR 4996 (*p* < 0.001), followed by an increase to EUR 6630 in 2015 (*p* < 0.01).

### Insurance

Overall, 10-year follow-up costs decreased from 2000 to 2015 (Fig. 1c). Follow-up costs after radiotherapy of SGCT stage I initially rose from EUR 1560 in 2000 to EUR 2347 in 2008 and clearly declined to EUR 759 until 2015. Costs for SHI and PHI follow the same underlying pattern. However, the cost reduction was more drastic for the statutory relative to the private insurance. Compared to 2000, statutory insurance costs across all cancer entities declined by 29% and 81% until 2008 and 2015, respectively. Yet, private costs only shrunk by 19% and 77% until 2008 and 2015, respectively. Consequently, costs were 2.2× (2000), 2.5× (2008), and 2.6× (2015) higher for the private compared to the statutory insurance. During the examined period, the reimbursement catalog for private insurances was not updated.

### Societal

**Fig. 2:**
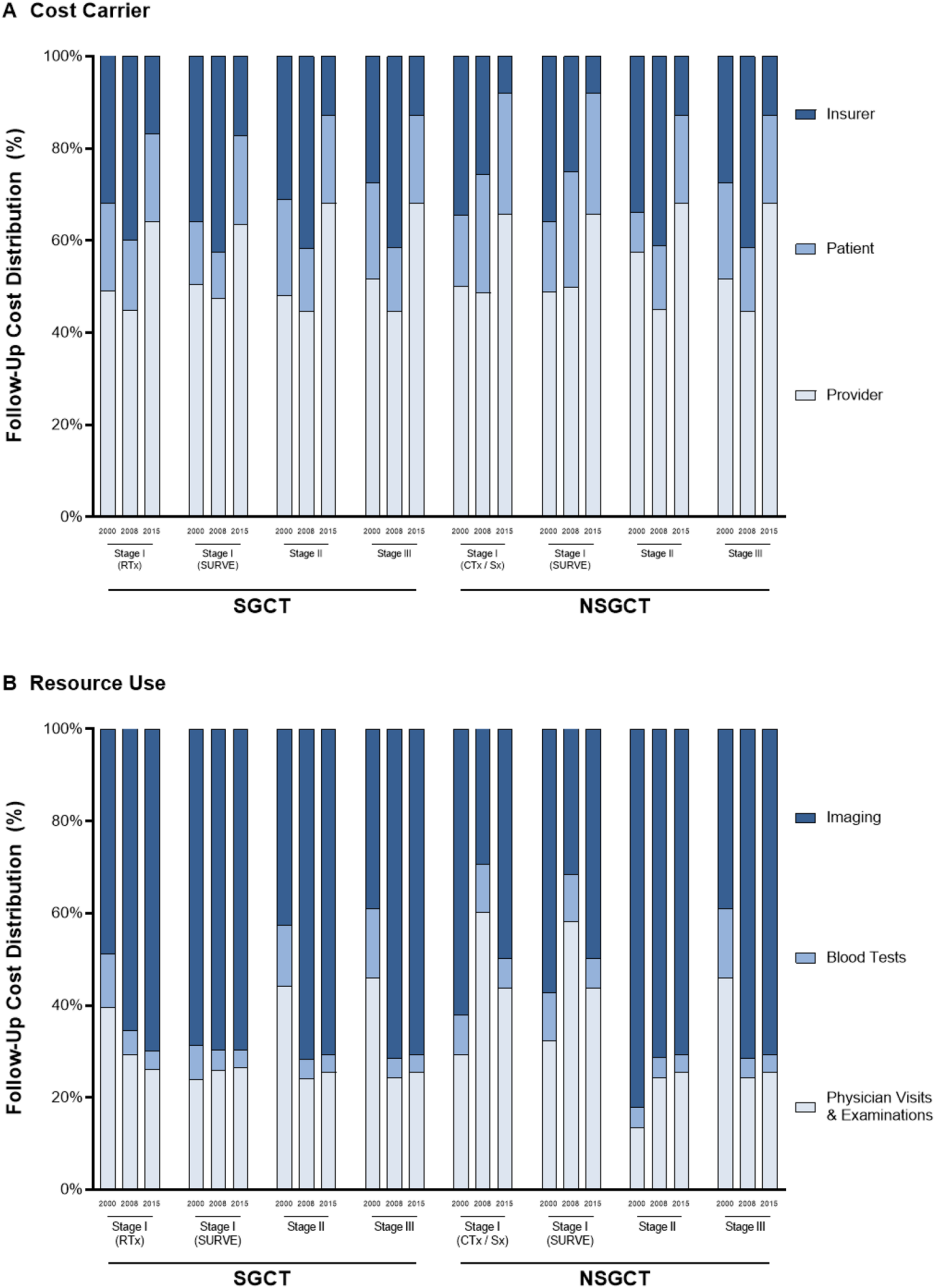
Testicular cancer follow-up cost distribution by cancer stages for **a** cost carrier and **b** resource use. *SGCT* seminomatous germ-cell tumor, *NSGCT* non-seminomatous germ-cell tumor, *RTx* radiotherapy, *SURVE* surveillance, *Sx* surgery, *CTx* chemotherapy

Taking a societal perspective, overall 10-year follow-up costs decreased from 2000 to 2015 except for SGCT in stage II (Fig. 1d). Follow-up costs after treatment of SGCT in stage II surged from EUR 7159 (2008) to EUR 9724 (2015; *p* < 0.001). The decline in 2008 can mainly be attributed to the decreased costs for insurance funds (Fig. 2a). While insurance funds incurred approximately one third of follow-up costs in 2000, this share declined to approximately 13% in 2015. Consequently, most of the financial burden was shifted to providers and payers, who collectively incurred approximately 87% of follow-up costs in 2015.

Generally, the resource use differs between SGCT and NSGCT due to different follow-up guidelines (Fig. 2b). SGCT follow-up costs were predominantly caused by medical imaging, which accounted for half of the spending in 2000. Given the rising importance of x-rays, CT, ultrasound, and FDG-PET, the share of imaging consumed more than two-thirds of follow-up costs. Spending for stage I NSGCT follow-ups was equally devoted to medical imaging and physician visits entailing examinations and blood tests. In contrast, imaging was the leading cost driver for stage III NSGCT, accounting for 71% of expenditures.

**Fig. 3:**
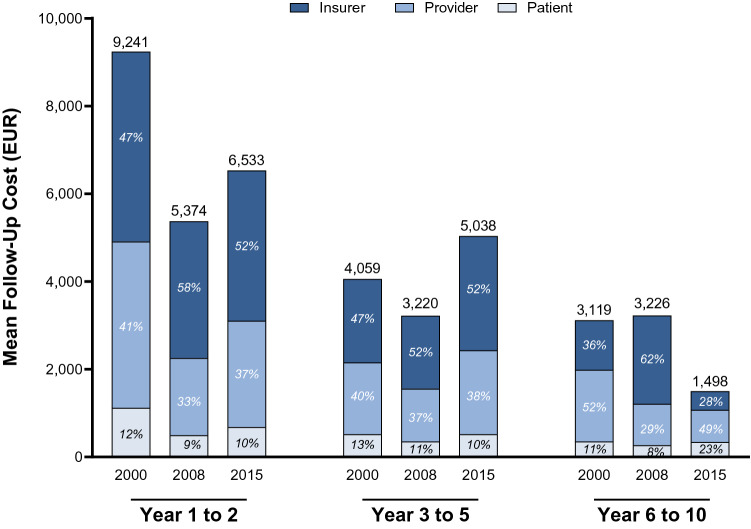
Mean follow-up cost for testicular cancer by follow-up year and perspective. All costs were inflation adjusted with the hospital and community health services index

In 2000, costs mainly accumulated during the first two years (EUR 9241) compared to the following three (EUR 4059) and five (EUR 3119) years (Fig. 3). Costs were redistributed over time, resulting in reductions of 29.1% during follow-up years 1–2 and of 52.3% during year 6–10 until 2015. Meanwhile, follow-up years 3 to 5 gained in importance as costs grew by 24.1%. This redistribution did not impact the expense burden borne by insurers (50%), providers (40%), and patients (10%) during follow-up years 1–5. After five years of follow-up costs were predominantly born by providers (52%) and insurers (36%) in 2000. However, guideline changes and reduced reimbursement rates shifted even more of the financial burden on patients (23%).

## Discussion

Cancer follow-up costs are relevant to assess the entire financial cancer burden on society. Therefore, costs incurred by all stakeholders—patients, providers, and insurers—were analyzed. Results underline that all three stakeholders face a significant economic barrier to testicular cancer follow-up. Consequently, the consideration of cancer follow-up costs is relevant and must be considered as an essential component of future clinical and health economic research. Precisely, cancer survivorship is an emerging financial and non-financial cost driver for the overall healthcare system, especially for patients. However, the overall financial burden depends on the cancer stage and the post-treatment “survivorship trajectory” (Lorgelly and Neri 2018). Simultaneously, there is a striking necessity for appropriate evidence to justify costs, radiation burden, and positive effect on mortality due to follow-ups (Park et al. 2018).

From a societal perspective follow-up costs decreased from 2000 to 2015 across most cancer stages—yet, costs were redistributed among stakeholders. In 2000, health insurances paid for one third of all follow-up costs. In 2015, insurances covered less than 13%. German statutory health insurers limited their cost exposure by the stepwise budgeting of medical services and lowering of reimbursement rates. Consequently, the financial burden of follow-up costs was gradually shifted to providers and patients.

Our results demonstrate that costs were particularly reduced during follow-up years five to ten and transferred to providers and patients. The increased economic burden to providers could especially disincentivize registered doctors in rural areas, with enhanced patient flows, to perform cancer follow-ups. Similarly, medical services and travel expenses not covered by health insurers discourage patient adherence to follow-ups. Research unveiled a low adherence to recommended follow-ups (Nielsen et al. 2015). Especially “perceived symptoms, motivation, affect, provider influences, readiness for medical follow-up, and knowledge of treatment exposures” influenced follow-up participation (Cox et al. 2009). In contrast, physicians only prepare detailed follow-up plans for less than one third of cancer survivors (Mayer et al. 2015; Sabatino et al. 2013). As a result, the current structure institutes financial and non-financial barriers for both providers and patients to adhere to cancer follow-up. Further research is necessary to analyze how such structural barriers impact follow-up adherence for both doctors and patients.

Recently, health policy concentrated on primary and tertiary prevention strategies. As a results, early detection and improved treatment options significantly reduced mortality and drove 10-year survival rates to 97% (Tarver 2012). The combination of increasing incidence and high cure rates effectively traps many cancer patients in the survivorship state for follow-ups. Many testicular cancer survivors experience long-term and late side effects of the treatment, especially after chemotherapy and radiotherapy (Chovanec et al. 2018). Secondary cancers and cardiovascular diseases represent the most serious threat to health after treatment. In addition, platinum-based chemotherapy might foster toxicity of the nerves and kidneys as well as hypogonadism (Haugnes et al. 2012). Cancer survivors were also shown to have a higher level of psychological disorders, e.g. depression or anxiety disorders (Kreiberg et al. 2020). These comorbidities impose a significant impact on the health-related quality of life after treatment.

Consequently, follow-up schedules should be adjusted to consider the growing number of survivors. There is an unmet need for more sophisticated follow-up patterns with a detailed match between evidence-based schedules and suitable patients to account for differential risk factors (Alfano et al. 2019). Therefore, personalized follow-up schedules could improve patient adherence, enhance survival, and decrease overall expenditure. Patients and providers are required to react to the observed financial shift by establishing innovative care concepts to significantly limit resource consumption. The careful design and implementation of structured disease management programs for cancer follow-ups offer the opportunity to enhance adherence to guidelines. Furthermore, structured programs might foster alignment of incentives across all stakeholders. Disease management programs might include in the first layer general practitioners (GP) and in the second layer urologists and oncologists.

Shared patient-centered survivorship trajectories between GPs, urologists and oncologist are necessary (De Padova et al. 2011). Yet, low-paid GP-led follow-ups are not a means to reduce overall healthcare spending, but one pathway to improve follow-up quality by personalizing care delivery. Outpatient cancer care delivery might be supplemented by virtual/online follow-ups—“e-oncology” (Bertucci et al. 2019). Thus, follow-ups could be (partly) substituted or complemented by specialist nurses, telephone consultations, or digital applications. In Germany, the Digital Healthcare Act (DVG), ratified in 2020, creates an official framework for reimbursement of digital applications (Dittrich et al. 2020). At the moment, Germany might not be able to provide the nationwide digital literacy and infrastructure for e-oncology. However, the introduction of such applications might intensify physician–patient relations, mitigate financial burdens faced by patients and providers, and erase inequalities in care quality and access by decreasing opportunity and travel costs for patients and providers.

### Strengths and limitations

Strengths of this study entail the comprehensive micro-costing method, wholistic approach to include all three perspectives, and assessment of costs for various treatment alternatives. First, follow-up spending was estimated by combining patient level data with EAU guidelines and reimbursement quotes. Second, direct and indirect medical costs were analyzed for insurers, providers, and patients. Third, relevant follow-up costs for different treatment options and cancer stages allow cost-effectiveness evaluations of testicular cancer treatment options. Results permit a detailed cost analysis across stakeholders, resource use, and follow-up year. Based on literature review, to the best of our knowledge, this is the first study assessing testicular cancer follow-up costs in Germany from 2000 to 2015.

However, the study is prone to some limitations. Firstly, follow-up costs are empirically determined and consequently not confirmed in clinical studies. Secondly, this health economic evaluation does not permit conclusions about the efficacy and cost-effectiveness of advances in diagnostic options and improved EAU guidelines. Thirdly, the underlying 100% “uptake” of guideline recommendations might not model reality. Further research is necessary to examine the follow-up costs of non-testicular cancer patients in and beyond Germany.

## Conclusion

Testicular cancer follow-ups encompass a considerable economic burden to all stakeholders. Follow-up costs differ depending on cancer histology, stage, and treatment strategy. Consequently, we proposed to consider follow-up expenses in cost-effectiveness evaluations of cancer treatments. Our results educate health care decision makers on the emerging economic burden shifted to patients and providers by statutory health insurers in Germany. The redistribution of the financial burden from insurers to patients and providers likely disincentivizes adherence to follow-up guidelines and consequently negatively influences health outcome—especially for the unprivileged population not covered by private insurance. Improved long-term care for cancer survivors, who are at risk for cardiovascular diseases, is urgently needed.
